# Robot-assisted radical resection for choledochal cysts in infants under three months of age: a multicenter retrospective study

**DOI:** 10.3389/fped.2026.1894326

**Published:** 2026-07-23

**Authors:** Lirong Zhang, Peng Song, Yunfeng Xiao, Quankun Zhou, Jian Yang, Libing Zhang, Zhou Jian Yang

**Affiliations:** 1 Mianyang Central Hospital, School of Medicine, University of Electronic Science and Technology of China, Mianyang, China; 2 Department of Cardiothoracic Surgery, Chengdu Women's and Children's Central Hospital, School of Medicine, University of Electronic Science and Technology of China, Chengdu, China

**Keywords:** congenital choledochal cyst, hepaticoenterostomy complications, laparoscopy, robotic surgery, young infant

## Abstract

**Background:**

To analyze the clinical efficacy and surgical experience of robot-assisted radical resection for choledochal cysts in infants under three months of age.

**Methods:**

This was a multicenter retrospective study conducted at the Department of Pediatric Surgery, Mianyang Central Hospital, and Chengdu Women's and Children's Central Hospital. A total of 31 patients diagnosed with congenital choledochal cysts under three months of age between May 2021 and May 2026 were included. The cohort comprised 9 boys and 22 girls, aged 10–86 days (median age: 52 days), with a body weight ranging from 3.0 to 6.5 kg (mean weight: 3.83 ± 0.75 kg). The primary clinical manifestation was obstructive jaundice. All patients underwent robot-assisted radical resection of choledochal cysts and were discharged after the resumption of feeding and the absence of postoperative complications.

**Results:**

Surgery was successfully completed in all 31 cases without conversion to open surgery. The operative time ranged from 173 to 239 min, with a median time of 190 min. All patients initiated feeding 24–48 h postoperatively, and No major early complications (e.g., bile leakage, pancreatitis, intestinal obstruction) occurred. Transient hyperbilirubinemia was observed in 12 patients (38.7%), which resolved with conservative management. The postoperative hospital stay ranged from 8 to 11 days, with a median stay of 9 days. All patients were followed up for 6–28 months. During the follow-up period, symptoms related to biliary obstruction resolved, and there were no occurrences of internal hernia, intestinal obstruction, or biliary stricture. All patients exhibited normal growth and development.

**Conclusion:**

For infants under three months of age with congenital choledochal cysts who present with surgical indications, robot-assisted radical resection appears safe and effective.This approach appears safe and feasible for carefully selected infants under three months of age. Favorable outcomes were observed, including successful completion of surgery without conversion, absence of major complications, and satisfactory cosmetic results. Further comparative studies are warranted to establish its role relative to laparoscopic surgery.

## Introduction

Congenital choledochal cyst (CCC) represents the most common congenital biliary malformation in the pediatric population, with an incidence of approximately 1 in 1,000 among Asian populations (1). The majority of affected infants are diagnosed via prenatal ultrasonography, while a minority present postnatally with clinical manifestations such as jaundice or vomiting, leading to definitive diagnosis through abdominal ultrasonography (2, 3). Historically, conventional open surgery constituted the standard therapeutic approach for CCC (3–5). In recent years, however, continuous advancements in minimally invasive surgical techniques have led to the widespread adoption of laparoscopic radical resection for choledochal cysts in clinical practice (6).

Nevertheless, the application of laparoscopic techniques remains technically challenging in neonates and young infants. This limitation is primarily attributed to the restricted intra-abdominal working space characteristic of this age group, which significantly increases the technical difficulty of the procedure (2). Furthermore, the steep learning curve associated with the technique demands a high level of surgical proficiency from the operator (5).

Robotic surgical systems offer distinct advantages, including tremor filtration, three-dimensional magnified visualization, and flexible, precise articulation of the robotic arms. These features facilitate the performance of refined and complex surgical maneuvers within confined anatomical spaces (1). Currently, multiple studies have established the feasibility and safety of robot-assisted laparoscopic radical resection for choledochal cysts in children and infants (2, 7). However, clinical reports regarding the application of this procedure in infants younger than three months of age remain scarce. Existing literature lacks a systematic and in-depth investigation into the technical nuances, standardized perioperative management, and short- and long-term clinical outcomes specific to this patient population.

This study retrospectively analyzed the complete clinical data of 31 patients with congenital choledochal cysts under three months of age who underwent robot-assisted radical resection at Mianyang Central Hospital and Chengdu Women's and Children's Central Hospital between May 2021 and May 2026. The aim of this study was to further evaluate the clinical feasibility, safety, and application value of this minimally invasive surgical approach in very young infants, thereby providing clinical evidence to optimize treatment strategies and improve patient prognosis.

## Clinical Data and Methods

### General data

A total of 31 infants with congenital choledochal cysts aged under three months were included in this study, comprising 9 males and 22 females. The age at surgery ranged from 10 to 86 days, and the body weight ranged from 3.0 to 6.5 kg. Preoperative imaging and clinical data indicated that 28 cases were diagnosed with extrahepatic biliary cysts via prenatal ultrasonography. Postnatal clinical manifestations were predominantly symptoms of biliary obstruction, including obstructive jaundice in 25 cases, clay-colored stools in 16, darkened urine in 28, a right upper quadrant mass or abdominal distention in 19, feeding difficulties or refusal to feed in 17, and irritability or frequent crying due to abdominal pain in 12. All infants received a confirmed diagnosis of congenital choledochal cyst via biliary ultrasonography, upper abdominal CT, and magnetic resonance cholangiopancreatography (MRCP). The maximum cyst diameter ranged from 19 to 63 mm, with a median diameter of 42 mm (Table 1).

**Table 1:** Demographic and clinical characteristics of 31 infants with choledochal cysts.

Variables	Data (*n* = 31)	95% CI
Gender (Male/Female)	9/22	
Age (days)	Median (Range): 52 (10–86)	43.1–59.5
Body weight (kg)	Mean ± SD: 3.83 ± 0.75 (Range: 3.0–6.5)	3.56–4.10
Cyst diameter (mm)	Median (Range): 42 (19–63)	37.9–47.1
Symptoms at presentation, n		-
Jaundice	25	-
Clay-colored stools	16	-
Feeding difficulties or refusal	17	-
Irritability or excessive crying	12	-
Right upper quadrant mass and/or distension	19	-
Operative time (min)	Median (Range): 190 (173–239)	185.0–197.0
Drainage tube duration (days)	Median (Range): 4 (3–5)	3.5–4.2
Time to resumption of diet (days)	Mean ± SD: 3.32 ± 0.93	2.99, 3.65
Postoperative hospital stay (days)	Median (Range): 9 (8–11)	8.5–9.4
Postoperative complications, n		-
Vomiting	0	-
Wound infection	0	-
Mucosal perforation	0	-
Incisional omental hernia	0	-
transient hyperbilirubinemia	12	-

**Inclusion Criteria:** Infants meeting any of the following surgical indications were included: 1) Imaging evidence of progressive enlargement of the choledochal cyst; 2) Clear manifestations of biliary obstruction with a direct bilirubin level ≥ 1.0 mg/dL or the presence of clay-colored stools, showing no symptomatic improvement after 2 weeks of oral ursodeoxycholic acid treatment.

**Exclusion Criteria:** 1) Patients complicated with acute biliary infection in the acute phase; 2) Patients with cardiopulmonary dysfunction unable to tolerate general anesthesia and surgery; 3) Patients with other severe congenital malformations.

This study protocol was approved by the Ethics Committee of Mianyang Central Hospital (Approval No. 2025KY-LW065). A waiver of individual research-specific informed consent was granted by the committee due to the retrospective nature of the study. All patients underwent standard surgical informed consent prior to the operation, as per routine clinical practice.

### Surgical methods

This was a multicenter retrospective study. All cases were sourced from Chengdu Women's and Children's Central Hospital (utilizing the Da Vinci surgical system) and Mianyang Central Hospital (utilizing the Toumai surgical system). All procedures were performed by senior attending surgeons or chief physicians. Preoperative bowel preparation was administered to all infants. Fasting protocols included 4 h for formula milk, 3 h for breast milk, and 2 h for clear fluids before surgery.

**Figure 1:**
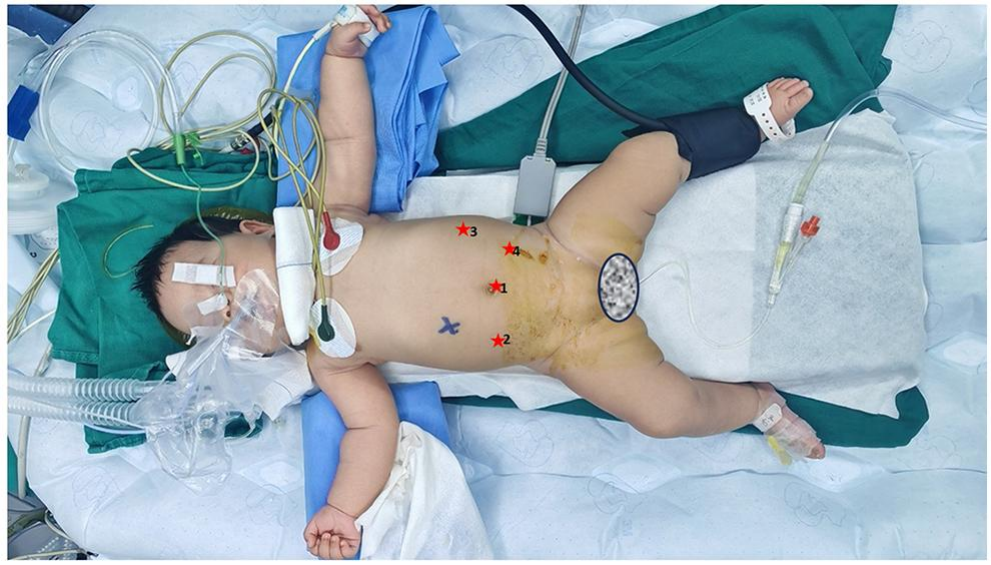
Patient positioning and port placement. The patient's torso is elevated by 20 cm to ensure sufficient working space for the robotic arms. (1): 3D laparoscope (umbilical port). (2): 8 mm right robotic port (anterior axillary line, umbilical level). (3): 8 mm left robotic port (between anterior axillary and midclavicular lines, slightly above umbilical plane). (4): 5 mm assistant port (between left robotic port and umbilicus).

After admission to the operating room, general anesthesia with endotracheal intubation was performed, and routine gastric and urinary catheters were placed. The patient was positioned supine with the torso elevated by 20 cm and the head of the bed raised by 15°. A robotic 3D laparoscope was inserted via an umbilical port. Two 8-mm robotic-specific trocars were placed in the right abdominal wall at the level of the umbilicus and in the left abdominal wall slightly above the umbilical plane, respectively. Care was taken to ensure an inter-port distance of > 3 cm and a distance of ≥ 4 cm from the operating ports to the hilar operative area (Figure 1).

**Figure 2:**
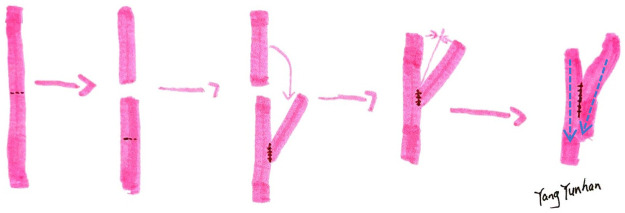
Schematic diagram of anti-reflux valve, arrows in the final schematic denote the flow direction of digestive fluids.

The robotic arms were not yet connected. Using conventional laparoscopic atraumatic forceps, the jejunum 15–20 cm distal to the ligament of Treitz was grasped. Pneumoperitoneum was then established, the umbilical trocar was removed, and the umbilical incision was extended longitudinally by approximately 2 cm. The distal jejunum was exteriorized, and mesenteric vessels were dissected and ligated. The jejunum was transected 15–20 cm from the ligament of Treitz, preserving a biliary limb (Roux-en-Y limb) of approximately 15–20 cm. An end-to-side anastomosis was performed between the proximal and distal jejunum to construct a rectangular anti-reflux valve. After closing the distal intestinal stump, the bowel was returned to the abdominal cavity (5, 8–10) (Figure 2).

**Figure 3:**
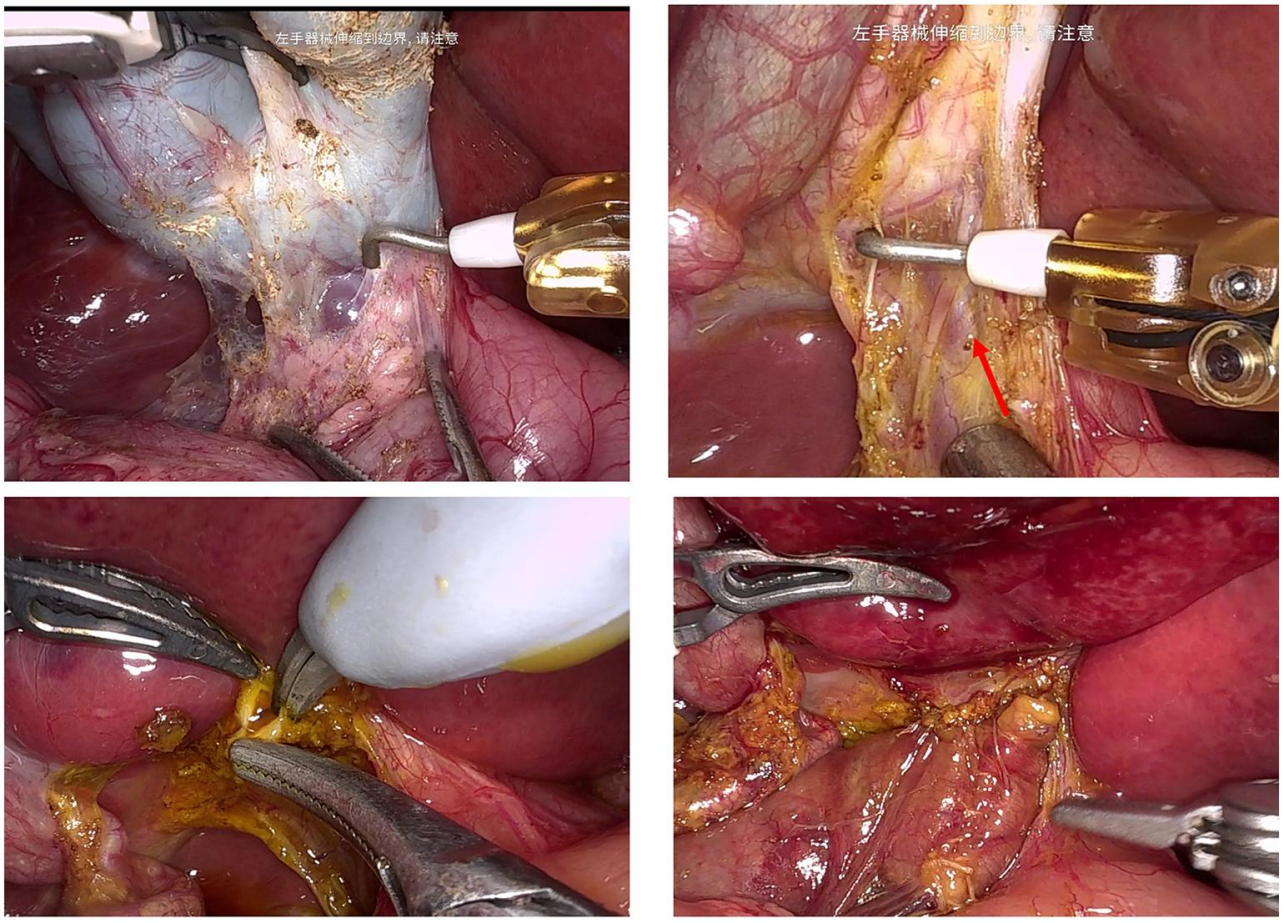
Key steps of cyst dissection and hepaticojejunostomy. **(A)** Dissection of the cyst towards the distal end. **(B)** Dissection towards the proximal end, revealing the portal vein posteriorly (indicated by the arrow). **(C)** Widening of the common hepatic duct opening to prevent postoperative stricture. **(D)** Anastomotic site after completion of hepaticojejunostomy, demonstrating satisfactory apposition of the bile duct and jejunal mucosa with intact serosal integrity.

The umbilical incision was reduced, the robotic 3D laparoscope was reinserted, and the robotic arm system was docked. The hilar anatomy was thoroughly explored. Percutaneous suspension of the gallbladder and the round ligament of the liver was performed to fully expose the common bile duct cyst. A 5-mm auxiliary instrument was placed in the left lower abdomen. With the round ligament and gallbladder suspended, dissection proceeded gradually from the distal end of the cyst. The cystic tissue was transected distal to the biliary stricture. Meticulous dissection was performed along the cyst wall toward the hepatic hilum until the non-dilated common hepatic duct was reached, which was then transected obliquely. The transverse colon was gently lifted, and a mesenteric tunnel was created just to the right of the hepatic hilum. The preserved biliary limb was pulled through the tunnel into the hilar field. The proximal end of the biliary limb was incised appropriately to create an anastomotic stoma slightly larger than the opening of the common hepatic duct. A 6–0 absorbable suture was used for a layered suture from left to right. The posterior wall was sutured first, followed by the anterior wall, meeting at the left side to complete the hepaticojejunostomy (Figure 3).

After confirming no active bleeding or bile leakage, the gallbladder was completely resected. A No. 10 abdominal drainage tube was placed in the operative area and extruded through the right abdominal wall port for fixation. Postoperatively, third-generation cephalosporins were routinely administered for 72 h to prevent infection. Oral intake of water was gradually initiated 24–48 h after surgery, followed by the progressive resumption of breastfeeding or formula feeding. For infants with elevated postoperative bilirubin levels, symptomatic treatment was administered with oral ursodeoxycholic acid (10 mg/kg once nightly). The abdominal drainage tube was removed when the drainage volume was < 10 mL/day.

### Statistical analysis

Continuous variables were expressed as mean ± standard deviation (SD) or median (range), as appropriate. Normality was assessed using the Shapiro–Wilk test. For normally distributed variables, 95% confidence intervals (CIs) for the mean were calculated using the standard formula (mean ± 1.96 × SEM). For non-normally distributed or discrete variables, 95% CIs for the median were calculated using the bootstrap method (1,000 resamples). Statistical analyses were performed using SPSS version 26.0 (IBM Corp., Armonk, NY, USA).

## Results

Robot-assisted radical resection of choledochal cysts was successfully completed in all 31 cases. No conversion to conventional laparoscopy or open surgery was required, and no intraoperative blood transfusions were necessary. Postoperatively, Clinical jaundice (visible icterus) resolved within 1 week postoperatively in all 25 patients who presented with preoperative jaundice. However, 12 patients had biochemical hyperbilirubinemia (elevated serum bilirubin levels) persisting for 1 week to 3 months, which was managed with oral ursodeoxycholic acid.

**Figure 4:**
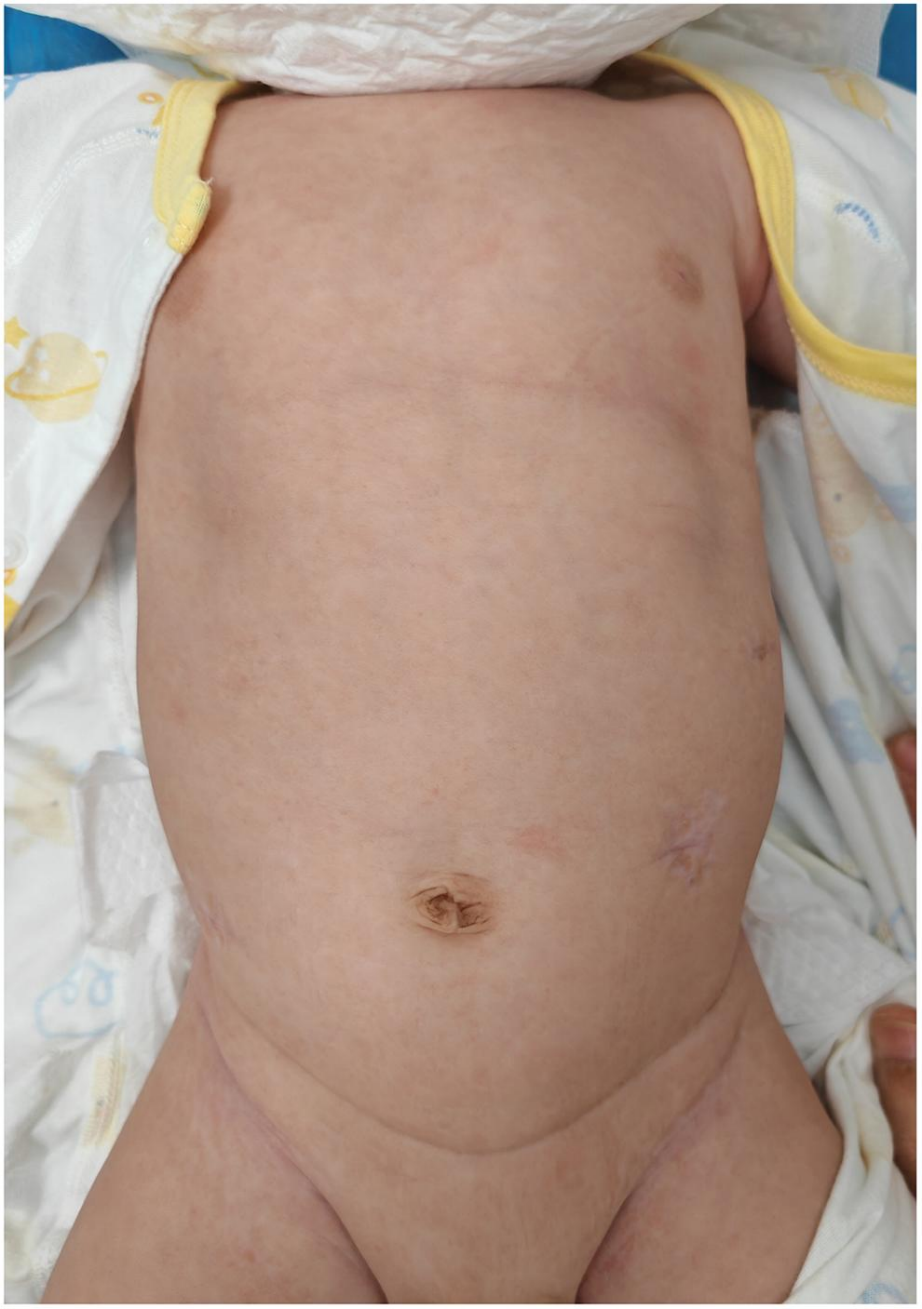
Postoperative cosmetic outcome. Abdominal appearance at 3 months after surgery demonstrates satisfactory healing with no obvious surgical scars.

No early postoperative complications, such as acute cholangitis, internal hernia, abdominal bleeding, bile leakage, or pancreatic fistula, occurred in any patient. The postoperative follow-up period ranged from 6 to 28 months. All patients demonstrated good healing of the abdominal wall incisions with high cosmetic satisfaction (Figure 4). During the follow-up period, no long-term complications, such as cholangitis, intestinal obstruction, or biliary stricture, were observed. all patients were reported to be following age-appropriate growth trajectories during follow-up visits, with no abnormalities in growth or development documented. Of the 31 patients, 19 (61.3%) completed at least 12 months of follow-up (median follow-up in this subgroup: 18 months; range: 12–28 months). No late complications (anastomotic stricture, cholangitis, or reoperation) were observed in this subgroup. No patients were lost to follow-up during the study period.

## Discussion

Congenital choledochal cyst (CCC) is the most common congenital biliary malformation in children. Currently, minimally invasive laparoscopic surgery is a mature and widely accepted treatment for this condition. Previous studies have suggested that robot-assisted surgery may be associated with shorter operative duration and hospital stay, as well as a lower complication rate, compared with traditional laparoscopic surgery (6, 8). However, clinical research and case reports regarding robot-assisted radical resection for choledochal cysts in infants under three months of age remain scarce both domestically and internationally (11). This study further explored the safety and technical nuances of this procedure in this specific age group.

Data from prior studies reveal notable drawbacks of laparoscopic management of CCC (12). Murali et al. (13) reported 28 cases of laparoscopic choledochal cyst resection, noting one conversion to open surgery, three cases of bile leakage, five cases of severe postoperative abdominal pain, and two unplanned readmissions (including one reoperation), underscoring the constraints of traditional laparoscopy in young patients. A large case-control study by Zhang et al. (6) demonstrated that the overall complication rate of robotic surgery was lower than that of laparoscopic surgery. Specifically, in infants with a common hepatic duct diameter < 5 mm, the incidence of bile leakage and anastomotic stricture was significantly lower in the robotic group, fully reflecting the unique advantages of robotic precision manipulation. Ishii et al. reported that robotic surgery was associated with fewer complications compared with laparoscopy in patients <10 kg. While their study included a broader age range, the shared focus on low-weight patients provides relevant context for our cohort, in which all patients weighed ≤6.5 kg (14). In this study, All procedures were completed successfully without conversion to open surgery or severe postoperative complications. Postoperative liver function indices improved significantly compared to preoperative levels, and symptoms of biliary obstruction such as jaundice and clay-colored stools completely resolved, further verifying the feasibility and safety of robotic surgery in extremely young infants under three months of age.

The core pathological change of choledochal cyst is congenital obstruction of the extrahepatic bile duct, which can cause continuous damage to liver function and carries a risk of biliary rupture (4, 19). Therefore, for infants with clear symptoms of biliary obstruction and liver function impairment, early surgical intervention is clinically recommended to promptly relieve the obstruction, prevent continuous liver damage, and avoid serious long-term complications (18). The anatomical space at the hepatic hilum in young infants is narrow, and the bile ducts are fine and adjacent to vital structures such as the portal vein and pancreas, requiring extreme precision in dissection and anastomosis. The 3D stereoscopic vision, ultra-high-definition magnified field of view, and tremor filtration system of the robotic system maximize the reduction of intraoperative deviations. This enables refined dissection of the common bile duct and precise differentiation of the biliary duct from surrounding blood vessels and pancreatic tissues, effectively reducing iatrogenic injury (2, 7, 19). In this study, there were no complications such as portal vein injury, pancreatic fistula, or active bleeding, and fine bile duct structures were dissected with precision. This fully corroborates the unique value of the robotic system in delicate anatomical manipulations in young patients, an advantage that has also been validated in other refined minimally invasive procedures such as pediatric Hirschsprung's disease (20, 21). Furthermore, the robotic surgical instruments and camera are controlled by the primary surgeon, avoiding issues of poor coordination between the surgeon and assistant and instrument interference common in traditional laparoscopy, thereby enhancing surgical fluency and stability.

Based on the surgical experience of these 31 extremely young infants, this study summarizes the technical points and core perioperative strategies for robot-assisted radical resection of choledochal cysts adapted for infants under three months of age. First, a standardized and individualized trocar placement scheme is essential. Given the extremely limited intra-abdominal working space in young infants, rational trocar placement is key to avoiding instrument interference and ensuring smooth operation. This study utilized a layout consisting of an umbilical main operating port combined with bilateral abdominal wall operating ports. The right port was placed at the anterior axillary line at the umbilical level; the left port was located between the anterior axillary line and the midclavicular line, slightly above the umbilical plane; and the assistant port was placed between the left operating port and the umbilicus. Strictly ensuring an inter-port distance of > 3 cm and a distance of ≥ 4 cm from the operating ports to the hilar surgical area effectively resolved the issue of instrument interference in a confined space. Second, the surgical workflow was optimized by performing jejunal mobilization and jejunojejunostomy first, followed by cyst excision and hepaticojejunostomy. This effectively shortened the docking time of the robotic arms and improved overall surgical efficiency. Third, the biliary enteric anastomosis was standardized using a right-to-left suture sequence. This conforms to the surgeon's conventional operating habits, facilitates exposure of the hilar view, and improves anastomotic precision. Fourth, addressing the characteristic fineness of the common hepatic duct in young infants, the duct was transected obliquely; for ducts with extremely small diameters, the proximal end was appropriately incised to enlarge the anastomotic stoma, thereby minimizing the risk of postoperative anastomotic stricture. Fifth, adherence to the principle of precise minimally invasive surgery involved dissecting closely along the biliary tissue to reduce unnecessary stripping and injury to surrounding normal tissues. Sixth, the rational use of suspension techniques for the round ligament and gallbladder provided full exposure of the hilar field, creating favorable conditions for refined manipulation.

Concurrently, this study summarizes the core characteristics and modifications of robotic surgery in young infants. First, the minimally invasive and cosmetic advantages are significant. The surgical trauma is minimal, and postoperative pain reactions are mild. Combined with the strong skin repair ability of infants, the postoperative abdominal wall scars are inconspicuous, meeting the cosmetic demands of families. Second, the approach adapts to the anatomical characteristics of young infants. The biliary ducts in young infants often lack recurrent inflammatory adhesions, and tissue layers are clear. This reduces the need for assistant involvement, requiring only basic assistance such as suture passing and cutting, which further highlights the precision advantage of the primary robotic surgeon. Third, the approach specifically addresses surgical difficulties in young infants. To address the issues of narrow body width and poor adaptability to surgical positioning, the patient was elevated by 20 cm intraoperatively. This prevented the robotic arms from being restricted by the operating table plane and ensured the range of motion and angles of the arms. There were no serious complications such as anastomotic leakage, anastomotic stricture, or portal vein injury in this group, fully confirming the safety and practicality of this set of surgical strategies and protocols.

This study has certain limitations. First, Due to the limited sample size and the absence of a control group, direct comparative analysis between surgical modalities and multivariate analysis for risk factors of complications were not feasible. Larger, multicenter studies are warranted to further validate these findings and explore potential subgroup differences. Second, although all patients completed at least 6 months of follow-up and 61.3% completed ≥12 months, the median follow-up of 9 months is insufficient to fully evaluate long-term outcomes such as late anastomotic stricture or cholangitis. Extended follow-up with longer observation periods is planned for future studies. Additionally, robot-assisted surgery has objective shortcomings, such as higher medical costs and a lack of specialized fine instruments adapted for extremely young infants, which still require clinical optimization and improvement. The use of two different robotic systems may introduce unmeasured heterogeneity, although no significant differences in perioperative outcomes were observed between the two groups in this series.

In conclusion, for infants under three months of age with congenital choledochal cyst who exhibit surgical indications, robot-assisted radical resection should be performed as early as possible under the premise of complete anesthesia, surgical, and perioperative monitoring conditions. This approach facilitates the timely relief of biliary obstruction and the protection of liver function. Compared with traditional laparoscopic surgery, robotic surgery demonstrates prominent advantages in refined anatomical dissection, precise anastomosis, complication reduction, rapid postoperative recovery, and cosmetic outcomes. It may serve as a viable minimally invasive option for carefully selected infants. Future prospective comparative studies are needed to confirm these preliminary findings (19–21).

## Data Availability

The raw data supporting the conclusions of this article will be made available by the authors, without undue reservation.
